# The association between dietary fiber intake and cognitive function: mediating role of inflammatory markers

**DOI:** 10.3389/fnut.2025.1638315

**Published:** 2025-09-29

**Authors:** Kaiyun Yan, Xinshuo Wang, Fengdan Wang, Baiyang Chen, Ziyu Zong, Jing Tian, Jing Zhao, Bo Li

**Affiliations:** School of Public Health, Jilin University, Changchun, China

**Keywords:** cognitive function, dietary fiber intake, inflammatory markers, NHANES, mediation analysis

## Abstract

**Aims:**

Cognitive impairment, frequently associated with neurodegenerative diseases such as Alzheimer's disease, may be associated with multiple factors including dietary fiber intake and inflammation. We aimed to explore the associations between reported dietary fiber intake, three novel inflammatory markers, and cognitive function.

**Methods:**

This observational and exploratory cross-sectional study utilized the data from the 2011–2014 of the National Health and Nutrition Examination Survey (NHANES). Digit Symbol Substitution Test (DSST), Consortium to Establish a Registry for Alzheimer's Disease Word Learning (CERAD-WL), CERAD Delayed Recall (CERAD-DR), and Animal Fluency tests (AFT) were used to assess the cognitive function. Linear regression was conducted to explore the relationships between reported dietary fiber intake, three novel inflammatory markers [Albumin-to-alkaline phosphatase ratio (AAPR), Neutrophil-to-Albumin Ratio (NAR), and Systemic Inflammation Response Index (SIRI)] and cognitive function. Mediation analysis was performed to identify the mediating role of inflammatory markers in the relationship between reported dietary fiber intake and cognitive function.

**Results:**

The final analysis included 2,461 participants. Reported dietary fiber intake was associated with CERAD-WL (β = 0.042, 95% CI = 0.018 to 0.066), AFT (β = 0.060, 95% CI = 0.020 to 0.100) and inflammatory markers (AAPR: β = 0.003, 95% CI=0.002 to 0.004; NAR: β = −0.003, 95% CI = −0.006 to −0.001; SIRI: β = −0.008, 95% CI = −0.015 to −0.001). AAPR was positively associated with WL (β = 1.184, 95% CI = 0.165 to 2.204) and AFT (β = 1.747, 95% CI = 0.229 to 3.264). AAPR mediated the positive association between reported dietary fiber intake and AFT, with mediation proportion of 17.88%.

**Conclusions:**

Reported dietary fiber intake, inflammatory markers, and cognitive function were pairwise associated. The AAPR played a mediating role in the association between reported dietary fiber intake and cognitive function.

## 1 Introduction

Cognitive impairment is an age-related state that occurs when various morphological, biochemical, metabolic, and circulatory changes caused by brain aging exceed a certain threshold (1). Cognitive impairment, the hallmark feature of dementia, is a severe neurological syndrome. Global projections indicate that 152.8 million people were affected by dementia worldwide in 2019 (2). Mild cognitive impairment (MCI) is often seen as an early indicator of Alzheimer's disease (AD) and is defined as a less severe stage of cognitive impairment, marked by slight alterations in memory and thinking skills (3). In older adults, dementia and cognitive impairment are the leading causes of long-term functional dependence and decline, leading to decreased quality of life, elevated healthcare expenses, and increased mortality rates (4). Cognitive impairment adversely influences an individual's capacity for daily living and imposes an economic burden on both society and families (5). Consequently, it is vital to recognize risk factors for cognitive impairment and inform the public about preventive measures to promote the cognitive health of older adults.

Previous studies have shown that cognitive impairment is associated with various factors such as dietary fiber intake, chronic low-grade inflammatory stress, sleep, and physical condition (6–9). Research has found higher dietary fiber intake is associated with improved specific components of cognitive function (10, 11). But some studies have also shown that dietary fiber intake is not associated with cognitive impairment in older adults (12). The effect of dietary fiber intake on cognitive performance in older adults is unclear, more research is needed to demonstrate this.

In addition to dietary factors, chronic inflammation in the body may cause morphological changes in the brain, damaging the normal function of specific areas of the brain, affecting cognitive function (13). The Albumin to Alkaline Phosphatase Ratio (AAPR), Neutrophil-Albumin Ratio (NAR), and Systemic Inflammatory Response Index (SIRI) are three novel inflammation-related biomarkers that reflect the systemic inflammatory state in the body and have been proven to be effective prognostic indicators for a range of diseases, particularly malignant tumors (14–16). The SIRI and AAPR were originally designed to predict the prognosis of digestive system cancer, and higher SIRI levels were associated with unfavorable prognostic outcomes (17, 18). Hooper et al. demonstrated that plasma alkaline phosphatase activity was significantly elevated in patients with AD and inversely correlated with cognitive function (19). In addition, some studies have confirmed that relatively low serum albumin concentration may be an independent risk factor for MCI in older adults (20). As a composite biomarker integrating both albumin and alkaline phosphatase, AAPR holds significant promise for assessing neuroinflammation-related cognitive decline. A study detected that NAR and SIRI levels were negatively related to the composite cognitive function score (Z-score) (21). NAR, as a comprehensive biomarker of neutrophils and albumin, represents the dual effects of neurotoxicity and protection in the acute phase, and can better reflect the degree of immune inflammation than a single indicator (22). Neutrophil count, lymphocyte count, and monocyte count are all closely related to immunity and inflammation. Given the critical role of inflammation in chronic diseases, hematologic markers readily available from routine blood tests were widely used to assess systemic inflammation (23). SIRI integrates the above three indicators, which can be used to distinguish the immune inflammatory response of three different pathways *in vivo*, and reflect the immune inflammatory state of the body more comprehensively (24). However, evidence on the relationships between inflammatory markers and cognitive function across various dimensions remains scarce. Recent evidence suggested that dietary fiber intake could also influence the systemic inflammation (25). A study by Saeed et al. identified that, in contrast to the high-fiber group, C-reactive protein (CRP) was higher in the low-fiber group (26). A cross-sectional study demonstrated that there was a significant negative correlation between dietary fiber intake and SIRI (27).

Recent studies have highlighted the ability of dietary interventions to influence inflammatory pathways associated with cognitive decline (28). For instance, high-fiber diets were associated with lower concentrations of pro-inflammatory cytokines, which were recognized for their role in neuroinflammation and consequent cognitive decline (29). Moreover, the Mediterranean diet, rich in dietary fiber, has demonstrated protective effects against cognitive decline, potentially through its anti-inflammatory properties (30). These findings underscore the importance of considering the impact of diet and inflammation on cognitive health when investigating older adults.

However, few studies have concentrated on the relationships between dietary fiber intake, inflammatory markers and cognitive function. Given the existing evidence regarding the potential advantages of fiber intake in improving inflammation levels and cognitive function, along with the influence of inflammation on cognitive performance, we hypothesized that the positive association between reported dietary fiber intake and cognitive function might be mediated through its anti-inflammatory effects. The objective of this research was to examine associations between reported dietary fiber intake, three inflammatory markers (AAPR, NAR and SIRI) and cognitive function and to determine the mediating role of inflammatory markers in the association of reported dietary fiber intake and cognitive function.

## 2 Materials and methods

### 2.1 Study population

**Figure 1:**
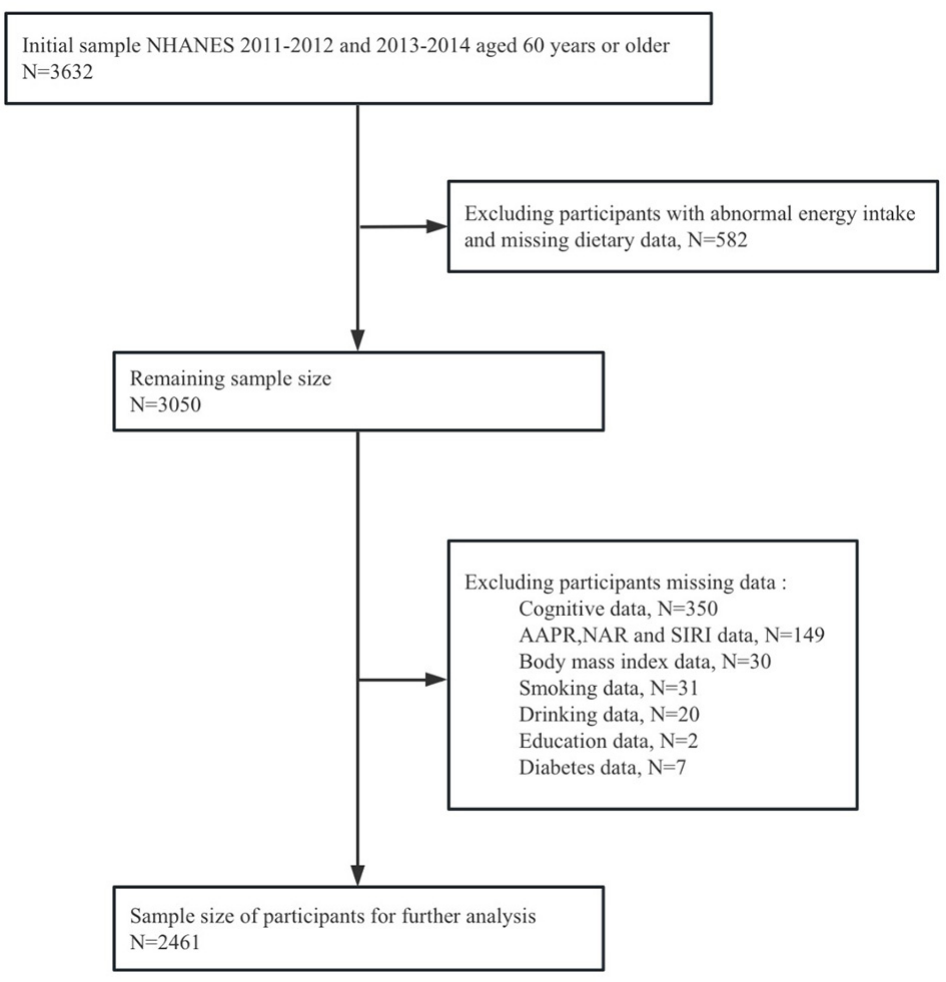
Flow chart portraying participant inclusion and exclusion in the current study.

This cross-sectional study utilized data from the National Health and Nutrition Examination Survey (NHANES), a national survey employing a complex, multistage, and probabilistic sampling design to evaluate the health status of U.S. adults. This program consists of five main data sets collected through interviews and physical examinations, including demographic information, diet, medical assessment, laboratory results and questionnaire data. Each participant completed the questionnaire and underwent examinations at the mobile examination center (MEC) over the course of 1 year, in order to obtain important research variables. The study included 3,632 adults aged 60 and above in NHANES from 2011 to 2014. Additionally, considering the complex sampling design and sample weights of NHANES, we removed missing values. 564 participants were excluded due to incomplete or unreliable 24-h recall data. Individuals who had extreme total energy intakes of < 500 or > 5,000 kcal/day for women (*n* = 13), and < 500 or > 8,000 kcal/day for men (*n* = 5) (31, 32) were further omitted. Participants with missing data on cognitive measures (*n* = 350), AAPR, NAR and SIRI (*n* = 149), and other covariates (*n* = 90) were excluded. Ultimately, a total of 2,461 participants were incorporated into this study, as detailed in Figure 1.

The NCHS ethics review board approved the NHANES study protocols, and all participants signed informed consent forms.

### 2.2 Reported dietary fiber intake

Participants underwent two interviews recalling dietary intake across the past 24-h, the initial one took place face-to-face at a mobile test center, while 3 to 10 days later, the second occurred over the phone. If dietary recall data for 2 days were available, the average was calculated. Otherwise, a single dietary recall was used (33).

### 2.3 Cognitive function

This study assessed cognitive performance by four cognitive tests: Digit Symbol Substitution Test (DSST), Consortium to Establish a Registry for Alzheimer's Disease Word Learning (CERAD-WL), CERAD Delayed Recall (CERAD-DR) and Animal Fluency tests (AFT) (34). The DSST was a paper-and-pencil cognitive test presented on a sheet of paper, which could be used to assess the reaction speed, long-term attention, and working memory of the subjects (35). The CERAD-WL requires participants to recall as many words as possible after reading 10 unrelated words aloud in various orders, for a total of 30 points. The CERAD-DR was performed after the AFT and DSST. Participants were asked to recall words from the CERAD-WL test, which was used to assess transient and delayed learning abilities. AFT was used to assess verbal fluency, in which participants were required to name as many animals as possible within 1 min, without any prompts. A Z-score was employed as a standardized measure of total cognitive performance to eliminate discrepancies in individual cognitive scores in this investigation. The Z-score was calculated as Z = (x-m)/σ, where x was the raw score, m was the population mean, and σ was the population SD. Composite Z-scores were created by summing the Z-scores of these four individual tests (DSST, AFT, CERAD-WL, CERAD-DR) (36). Z scores < -1 were characterized as “cognitive impairment” (37).

### 2.4 Definition of inflammatory markers (AAPR, NAR and SIRI)

Peripheral blood samples from NHANES participants were analyzed at a mobile Examination center (MEC) using a Beckman Coulter HMX hematology analyzer. Lymphocyte, neutrophil, and monocyte counts were measured by complete blood count. The Beckman UniCel^®^DxC800 Synchron analyzer was employed to assess serum ALP levels and serum albumin levels. Based on peripheral blood cell counts, AAPR, NAR, and SIRI are calculated as follows. AAPR = albumin (g/L)/alkaline phosphatase (U/L) (38), NAR = neutrophil count (10^9^/L)/albumin (g/dL) (39), SIRI = monocyte count (10^9^/L) × neutrophils count (10^9^/L)/lymphocyte count (10^9^/L) (40). The specific measures and detailed information can be found in the NHANES Laboratory/Medical Technician Procedures Manual (41).

### 2.5 Covariates

We adjusted covariates factors that might be associated with reported dietary fiber intake and cognitive function (including age, sex, race, education status, body mass index (BMI), smoking status, drinking status, energy intake (42) and history of diabetes). Smoking status was assessed as non-smokers (had smoked < 100 cigarettes in their entire life), former smoker (had smoked ≥ 100 cigarettes in their entire life and answered negatively to the question, “Do you smoke now?”), or current smoker (≥ 100 cigarettes in their entire life and answered affirmatively to the question, “Do you smoke now?”). Smoking status was categorized into “never smokers” and “ever smokers” by combining former smokers and current smokers (43). Drinking status was classified into never drinkers (no alcohol consumption throughout lifetime or in the past 12 months), former drinkers (prior alcohol consumption but no intake in the past 12 months), and current drinkers (consumption of ≥12 alcoholic drinks in the past year or in their lifetimes and at least one drinking day reported in the past year). The drinking status was later categorized as “never drinker” and “ever drinker” by combing former and current drinkers (44). BMI was categorized into three categories: underweight or healthy weight (BMI < 25.0 kg/m^2^), overweight (25.0 ≤ BMI < 30.0 kg/m^2^), and obese (≥ 30.0 kg/m^2^). Energy intake was categorized into three categories: low intake (500 to 1,600 per day for women, 500 to 2,000 calories per day for men), adequate intake (1,600 to 2,400 calories per day for women, 2,000 to 3,000 calories per day for men) and high intake (>2,400 calories per day for women and 3,000 calories per day for men) (45).

### 2.6 Statistical analysis

According to the analysis guidelines of NHANES, weighting was performed using strata, PSU and weight. The weight used was WTDRD1 or WTDR2D. All the statistical analysis was conducted under the instruction of NHANES complex sample weight. In this study, IBM SPSS24.0 software and R4.3.2 were used to analyze the data. Continuous and categorical data were characterized using medians (IQRs) or numbers (weighted proportions), respectively. Differences between groups in continuous and categorical variables were assessed by the Wilcoxon rank-sum test and chi-square test, respectively. Linear regression was conducted to explore the relationships between reported dietary fiber intake, inflammatory markers and cognitive function. The results were presented as regression coefficients (β) and 95% confidence intervals (CI). Model 1 was adjusted for sex, age, race, and education status, and model 2 further adjusted for BMI, smoking status, drinking status, energy intake and history of diabetes based on model 1. The multicollinearity of the independent variables was tested by calculating the variance inflation factor (VIF). The VIF values of all independent variables were < 5, indicating that there was no significant multicollinearity problem in the model. Finally, mediation analysis was performed by “mediation” packages to explore the mediating role of inflammatory markers in the relationship between reported dietary fiber intake and cognitive function. The bootstrap method was used to obtain the CI of the indirect effect. The proportion of the mediated effect was calculated using the following formula: (mediated effect/total effect) × 100%. Statistical significance was determined when the two-tailed *P*-value < 0.05. No multiple comparison correction was performed.

## 3 Results

### 3.1 General characteristics

Among the 2,461 participants involved in this study, 422 (9.9%) were assigned to the cognitively impaired group. As shown in Table 1, reported fiber intake and AAPR in cognitive impairment group were lower than that in non-cognitive impairment group, while NAR was higher in cognitive impairment group. SIRI had no differences between participants with and without cognitive impairment. Meanwhile, there were differences in age, race, education status, drinking status, history of diabetes and energy intake (*P* < 0.05).

**Table 1:** Characteristics of participants [median (IQR)/n (%)].

**Characteristics**	**Total *n* = 2,461**	**Non-cognitive impairment *n* = 2,039 (90.1%)**	**Cognitive impairment *n* = 422(9.9%)**	***Z/χ^2^***	***P*-value**
Fiber intake, g/d	16.05 (11.70,21.80)	16.30 (12.00,22.20)	13.50 (9.65,18.15)	6.330	< 0.001
AAPR	0.66 (0.54,0.81)	0.67 (0.55,0.82)	0.57 (0.45,0.73)	−5.792	< 0.001
NAR	0.93 (0.74,1.21)	0.93 (0.74,1.19)	1.02 (0.78,1.37)	−2.723	0.006
SIRI	1.17 (0.81,1.84)	1.17 (0.81,1.81)	1.26 (0.83,2.04)	−1.311	0.190
**Age, years**				96.054	< 0.001
60–70	1,463 (62.0)	1,289 (65.2)	174 (33.1)		
>70	998 (38.0)	720 (34.8)	248 (66.9)		
**Sex**				0.136	0.759
Male	1,223 (47.7)	983 (47.8)	240 (46.6)		
Female	1,238 (52.3)	1,056 (52.2)	182 (53.4)		
**Race**				126.956	< 0.001
Non-Hispanic White	1,245 (79.1)	1,114 (82.1)	131 (51.2)		
Other	1,216 (20.9)	925 (17.9)	291 (48.8)		
**Education status**				219.465	< 0.001
Below high school	586 (14.8)	342 (11.5)	244 (45.4)		
High school	581 (22.4)	491 (22.0)	90 (25.4)		
Above high school	1,294 (62.8)	1,206 (66.5)	88 (29.2)		
**BMI**				7.075	0.206
Under and healthy weight	648 (26.7)	527 (26.0)	121 (33.7)		
Overweight	873 (35.8)	726 (36.3)	147 (30.7)		
Obese	940 (37.5)	786 (37.7)	154 (35.6)		
**Smoking status**				0.196	0.627
Never smoker	1,215 (51.1)	1,014 (51.2)	201 (49.7)		
Ever smoker	1,246 (48.9)	1,025 (48.8)	221 (50.3)		
**Drinking status**				11.195	0.006
Never drinker	360 (12.4)	282 (11.7)	78 (19.1)		
Ever drinker	2,101 (87.6)	1,757 (88.3)	344 (80.9)		
**Diabetes**				26.182	< 0.001
No	1,787 (78.0)	1,528 (79.4)	259 (65.1)		
Yes	674 (22.0)	511 (20.6)	163 (34.9)		
**Energy intake**				−8.854	< 0.001
Low	1,282 (44.6)	981 (41.9)	301 (68.8)		
Adequate	979 (46.4)	870 (48.5)	109 (27.3)		
High	200 (9.0)	188 (9.6)	12 (3.9)		

AAPR, albumin to alkaline phosphatase ratio; NAR, neutrophil-to-albumin ratio; SIRI, systemic inflammation response index; BMI, Body Mass Index.

### 3.2 Correlations of reported dietary fiber intake with cognitive function and inflammatory markers

As shown in Table 2, higher reported dietary fiber intake was associated with higher cognitive function scores of CERAD-WL, DSST and AFT in the crude model. Reported dietary fiber intake remained a significant association with CERAD-WL (β = 0.042, 95% CI = 0.018 to 0.066) and AFT (β = 0.060, 95% CI = 0.020 to 0.100) in the fully adjusted model, which implied that increasing reported dietary fiber intake by 1 g/day increased CERAD-WL and AFT scores by 0.042 and 0.060 points, respectively.

**Table 2:** Linear regression model for reported dietary fiber intake on cognitive performance.

**Cognitive performance**	**β (95%CI)**	***P*-value**
**CERAD-WL**		
Crude model	0.053 (0.029, 0.076)	**< 0.001**
Model 1	0.051 (0.032, 0.071)	**< 0.001**
Model 2	0.042 (0.018, 0.066)	**0.001**
**CERAD-DR**		
Crude model	0.015 (0.000, 0.030)	0.055
Model 1	0.014 (−0.001, 0.028)	0.061
Model 2	0.010 (−0.004, 0.025)	0.157
**DSST**		
Crude model	0.195 (0.086, 0.304)	**0.001**
Model 1	0.125 (0.029, 0.220)	**0.012**
Model 2	0.103 (−0.005, 0.210)	0.061
**AFT**		
Crude model	0.097 (0.057, 0.138)	**< 0.001**
Model 1	0.072 (0.033, 0.111)	**0.001**
Model 2	0.060 (0.020, 0.100)	**0.005**

Crude model: no covariates adjusted.

Model 1 = Sex + Age + Race + Education status.

Model 2 = Model 1 + BMI + Smoking status + Drinking status + Diabetes+ Energy intake.

CERAD-WL, Consortium to Establish a Registry for Alzheimer's Disease Word Learning; CERAD-DR, CERAD Delayed Recall; DSST, Digit Symbol Substitution Test; AFT, Animal Fluency tests; BMI, Body Mass Index. The bold values represent statistically significant results.

Reported dietary fiber intake was associated with AAPR and NAR in the crude model. Similarly, reported dietary fiber intake was significantly associated with AAPR (β = 0.003, 95% CI = 0.002 to 0.004), NAR (β = −0.003, 95% CI = −0.006 to −0.001), and SIRI (β = −0.008, 95% CI = −0.015 to −0.001) in the fully adjusted model (Table 3), which implied that increasing reported dietary fiber intake by 1 g/day increased AAPR by 0.003 and decreased NAR and SIRI by 0.003 and 0.008, respectively.

**Table 3:** Linear regression model for reported dietary fiber intake on inflammatory markers.

**Inflammatory markers**	**β (95%CI)**	***P*-value**
**AAPR**		
Crude model	0.002 (0.001, 0.004)	**0.001**
Model 1	0.002 (0.000, 0.003)	**0.019**
Model 2	0.003 (0.002, 0.004)	**< 0.001**
**NAR**		
Crude model	−0.004 (−0.006, −0.002)	**< 0.001**
Model 1	−0.004 (−0.006, −0.002)	**< 0.001**
Model 2	−0.003 (−0.006, −0.001)	**0.013**
**SIRI**		
Crude model	−0.004 (−0.009, 0.002)	0.224
Model 1	−0.007 (−0.013, −0.001)	**0.025**
Model 2	−0.008 (−0.015, −0.001)	**0.018**

Crude model: no covariates adjusted.

Model 1 = Sex + Age + Race + Education status.

Model 2 = Model 1 + BMI + Smoking status + Drinking status + Diabetes+ Energy intake.

AAPR, albumin to alkaline phosphatase ratio; NAR, neutrophil-to-albumin ratio; SIRI, systemic inflammation response index; BMI, Body Mass Index. The bold values represent statistically significant results.

### 3.3 Associations between inflammatory markers and cognitive function

**Figure 2:**
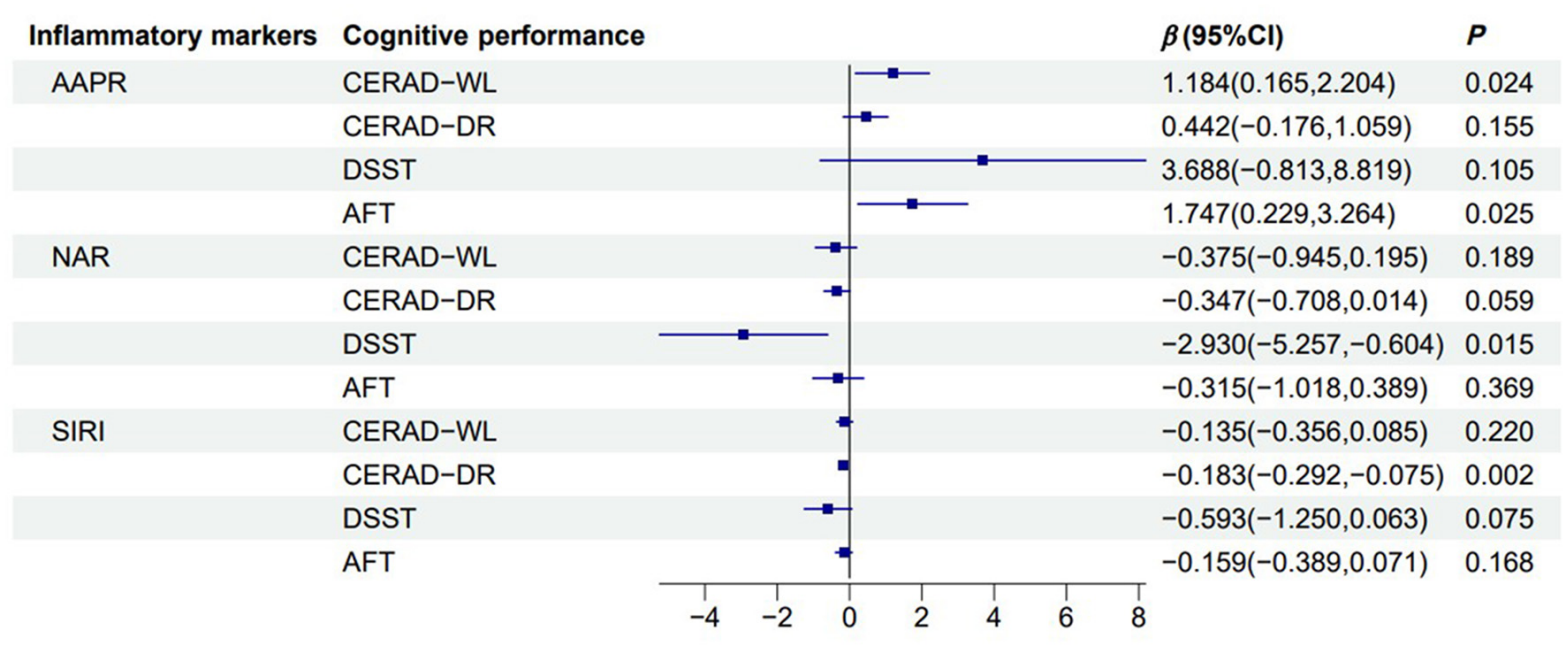
Forest plot of the association between inflammatory markers and cognitive function. Linear regression model illustrating the association between reported dietary fiber intake (g) and cognitive function across multiple cognitive assessments. The black solid line represents 0, the blue solid line represents the 95% confidence interval, and the blue square represents the estimated value (β). Adjusted for sex, age, race, education status, smoking status, drinking status, BMI, diabetes, energy intake. CERAD-WL, Consortium to Establish a Registry for Alzheimer's Disease Word Learning; CERAD-DR, CERAD Delayed Recall; DSST, Digit Symbol Substitution Test; AFT, Animal Fluency tests; AAPR, albumin to alkaline phosphatase ratio.

As shown in Figure 2, in model 2, AAPR was significantly positively associated with CERAD-WL (β = 1.184, 95% CI = 0.165 to 2.204) and AFT (β = 1.747, 95% CI = 0.229 to 3.264). NAR was significantly negatively associated with DSST (β = −2.930, 95% CI = −5.257 to −0.604), and SIRI was negatively associated with CERAD-DR (β = −0.183, 95% CI = −0.292 to −0.075).

### 3.4 Mediating effect of inflammatory markers on reported dietary fiber intake and cognitive function

**Figure 3:**
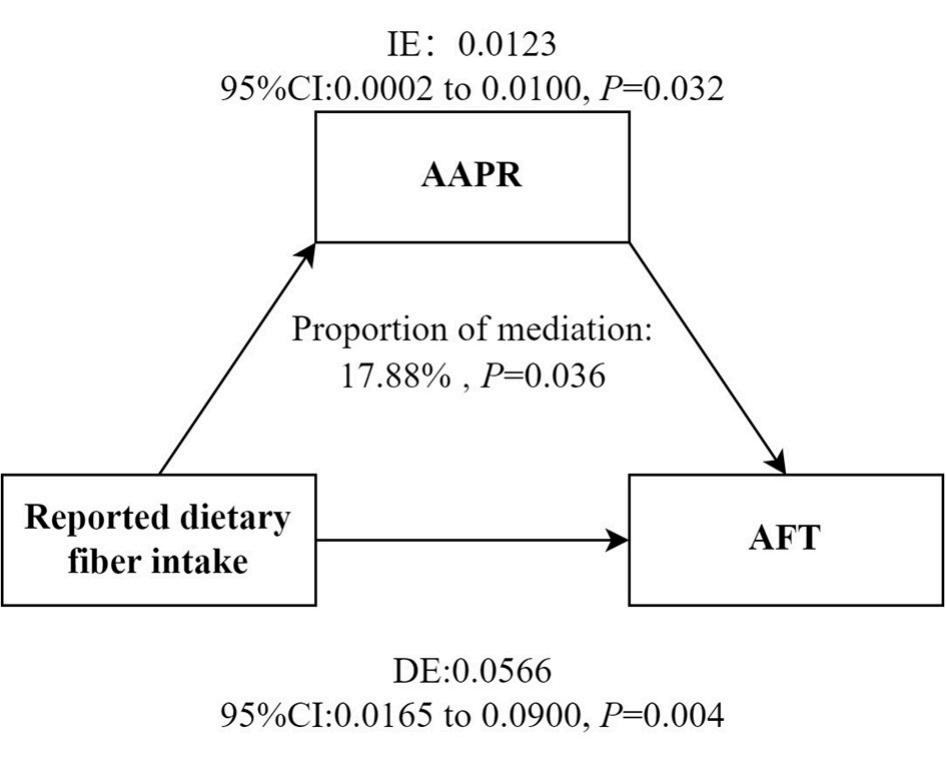
Mediation models. Figure demonstrating mediation models with independent variables of reported dietary fiber intake, mediators of AAPR and dependent variable of AFT. IE, Indirect effect; DE, Direct effect. Adjusted for sex, age, race, education status, smoking status, drinking status, BMI, diabetes and energy intake. AFT, Animal Fluency tests; AAPR, albumin to alkaline phosphatase ratio.

As shown in Table 4, the effects of reported dietary fiber intake on WL and AFT were reduced after adjusting for inflammatory markers. AAPR mediated the association between reported dietary fiber intake and AFT (proportion of medaiiton: 17.88%; indirect effect = 0.0123; 95% CI = 0.0002 to 0.0100; Figure 3). However, no mediating effect of AAPR was observed in the association between reported dietary fiber intake and CERAD-WL (Supplementary Figure 1).

**Table 4:** Linear regression model for reported dietary fiber intake and AAPR on CERAD-WL and AFT.

**Cognitive performance**	**Variables**	**β (95%CI)**	***P***
CERAD-WL	Reported dietary fiber intake	**0.039 (0.014, 0.064)**	**0.003**
	AAPR	**1.057 (0.035, 2.080)**	**0.043**
AFT	Reported dietary fiber intake	**0.055 (0.015, 0.095)**	**0.008**
	AAPR	**1.567 (0.057, 3.077)**	**0.042**

Adjusted for sex, age, race, education status, smoking status, drinking status, BMI, diabetes and energy intake.

CERAD-WL, Consortium to Establish a Registry for Alzheimer's Disease Word Learning; AFT, Animal Fluency tests; BMI, Body Mass Index; AAPR, albumin to alkaline phosphatase ratio.

The “AAPR” row represents adding the “AAPR” variable to the model. The bold values represent statistically significant results.

## 4 Discussion

This study demonstrated the association between higher reported dietary fiber intake and better cognitive performance assessed by CERAD-WL and AFT, and the association between higher AAPR and better cognitive performance measured by CERAD-WL and AFT also. In addition, AAPR played a mediating role in the relationship between reported dietary fiber intake and cognitive function, with a mediation proportion of 17.88%.

He et al. analyzed 2,713 older adults and found that dietary fiber had a significant positive correlation with AFT in the fourth quarter (42). Previous studies have demonstrated that fiber intake was positively associated with AFT and WL (46). These findings are consistent with our results, demonstrating that higher fiber intake is associated with better performance on both CERAD-WL and AFT. However, the relationship between fiber intake and DSST remains controversial. A study demonstrated a significant association between dietary fiber and the DSST in older adults, revealing an approximate linear dose-response relationship between dietary fiber intake and DSST (10). However, some previous studies found no association between dietary fiber intake and DSST (11), which was similar to our finding. More persuasive studies are needed to further explore the relationship of fiber with DSST. Future studies should employ longitudinal designs to clarify the temporal relationship between dietary fiber intake and specific cognitive domains, particularly DSST. The potential mechanisms underlying the observed association between dietary fiber consumption and lower cognitive decline have not been fully elucidated. The gut microbiome significantly affects brain function via the gut-brain axis (47). After reaching the colon, dietary fiber is fermented and metabolized by bacteria to produce short-chain fatty acids (SCFA) (48), exerting their metabolic benefits through gut-cranial neural circuits (49). SCFAs such as acetate, propionate, and butyrate regulate neuroinflammation and synaptic growth, survival, and differentiation (50). These interactions underscore the significant function of dietary fiber not just in digestive health but also in sustaining cognitive vitality through complex biological pathways.

Our findings suggested that dietary fiber may beneficially influence inflammatory level, while higher inflammation levels showed a strong association with poorer cognitive function. Dietary fiber plays an anti-inflammatory role by changing intestinal flora and producing short-chain fatty acids (51). Another study showed that dietary fiber could directly attenuate the production of inflammatory cytokines by dendritic cells co-cultured with intestinal epithelial supernatants through interaction with Toll-like receptors (52). Chronic inflammation is a key pathogenetic element that leads to the progression of AD and cognitive impairment. Chronic inflammation in the body may cause morphological changes in the brain and impair the normal function of specific brain regions (13). In addition, inflammation also disrupts the integrity of the blood-brain barrier (53), allowing inflammatory markers to penetrate and irritate nerves, resulting in neurodegeneration. Our findings revealed that AAPR partly mediated the association between fiber intake and cognitive function among older adults, accounting for 17.88% of the total effect. AAPR can be utilized to forecast the prognosis of cancer, such as lung cancer (54) and cholangiocarcinoma (55). Higher AAPR reflects elevated albumin and reduced ALP, potentially benefiting cognition through dual pathways: albumin maintains blood volume, regulates osmotic pressure, transports nutrients and metabolites, and acts as an antioxidant, all of which are beneficial to brain cells (56), while lower ALP may indicate reduced vascular inflammation. Maintaining a low level of ALP may exert a protective effect on cognitive function by inhibiting neuroinflammatory responses, without causing significant harm (57). High alkaline phosphatase (58) and low albumin were associated with high levels of inflammation (59). Approximately one-fifth of the dietary fiber-cognitive benefits may be achieved through this inflammatory pathway, while the remaining 80% of the effects may be mediated by other mechanisms such as the gut microbiota/short-chain fatty acids. Moreover, foods rich in fiber are usually rich in anti-inflammatory components such as whole grains, providing a theoretical basis for their substantive mediating role (60). It is suggested that decreased AAPR indicates the existence of different degrees of malnutrition and inflammation (61). Therefore, according to the Dietary Guidelines for Americans (2020–2025), for older adults whose fiber intake is below the recommended levels (< 22 grams per day for women and < 28 grams per day for men), increasing the fiber intake in their diet to meet these standards may help reduce systemic inflammation and may also lower the risk of cognitive decline.

This study has several notable advantages. Firstly, it was the first study exploring the mediating effect of AAPR on the relationship between dietary fiber intake and cognitive function. Secondly, our study was based on NHANES, a survey that represents the national population. However, there are some limitations in the present study. Firstly, as a cross-sectional study, causation cannot be inferred and there might be a reverse causal relationship. The effect values for some of the associations were relatively small. Future large-scale longitudinal or interventional studies, particularly randomized controlled trials (RCTs), are required to establish causation for the observed associations and to explore their underlying mechanisms. Secondly, the study population was located in the U.S., and the results may not be generalizable to other populations with different dietary habits, genetic backgrounds, or healthcare systems. Thirdly, although we adjusted for key confounders, residual confounding from unmeasured factors may still influence the observed associations. Future research should strive to collect more detailed data on these factors to better control these potential residual confounders. In addition, the assessment of reported dietary fiber intake relied on 24-h recall interviews, which may have been affected by recall bias. Finally, supplements were not considered for this study, which may result in inaccurate calculations of dietary fiber intake.

## 5 Conclusion

Reported dietary fiber intake, inflammatory markers, and cognitive function were pairwise associated. The AAPR played a mediating role in the association between reported dietary fiber intake and cognitive function.

## Data Availability

The original contributions presented in the study are included in the article/Supplementary material, further inquiries can be directed to the corresponding author.
