# Supplementary material for: The association between dietary fiber intake and cognitive function: mediating role of inflammatory markers
Source: Front Nutr. 2025 Sep 29;12:1638315. doi: 10.3389/fnut.2025.1638315 (PMC12515671; doi:10.3389/fnut.2025.1638315)
Supplement: Supplementary file 1 [file Supplementary_file_1.docx]

Supplementary Material


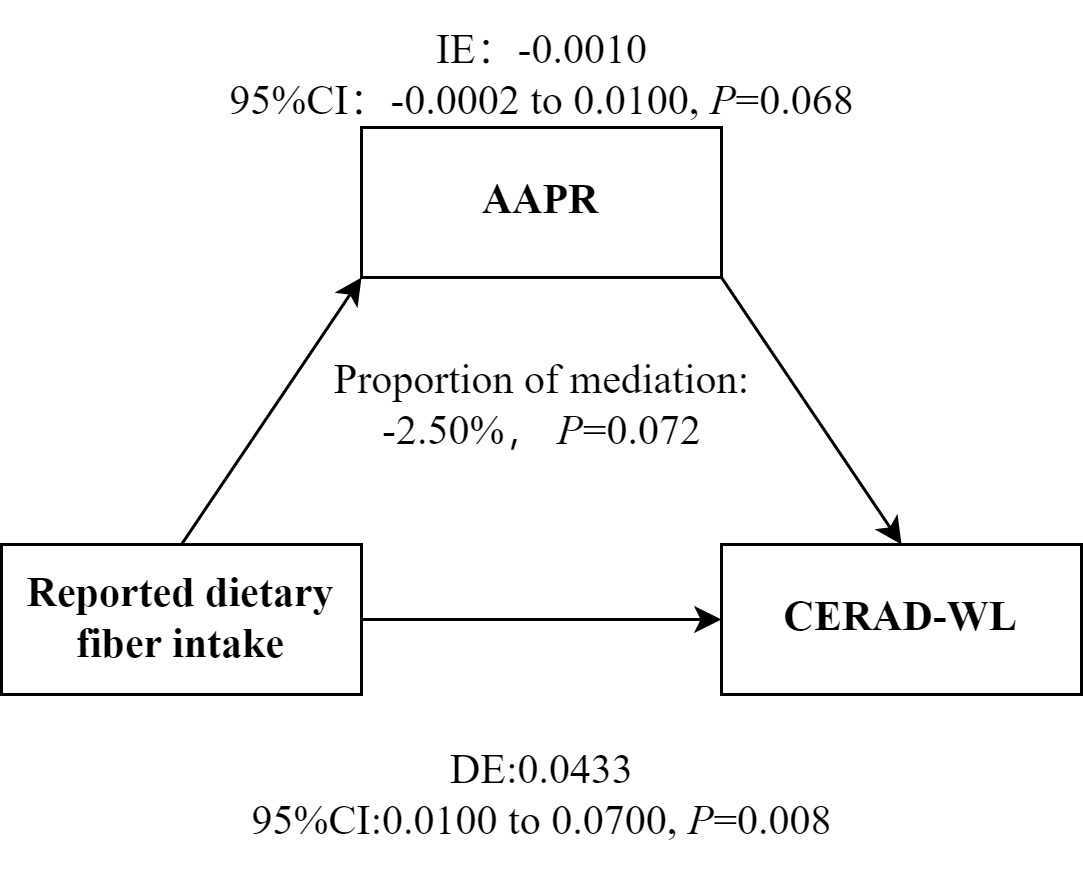


**Supplementary Figure 1.** Mediating effect of AAPR between reported dietary fiber intake and CERAD-WL
